# An investigation of horizontal transfer of feed introduced DNA to the aerobic microbiota of the gastrointestinal tract of rats

**DOI:** 10.1186/1756-0500-5-170

**Published:** 2012-04-01

**Authors:** Lise Nordgård, Lorenzo Brusetti, Noura Raddadi, Terje Traavik, Beate Averhoff, Kaare Magne Nielsen

**Affiliations:** 1 GenØk, Centre for Biosafety, Science Park, 9294 Tromsø, Norway; 2 Department of Pharmacy, Faculty of Medicine, University of Tromsø, N-9037 Tromsø, Norway; 3 Faculty of Science & Technology, Free University of Bozen/Bolzano, 39100 Bozen/Bolzano, Italy; 4 Department of Food Science, Technology and Microbiology, University of Milan, 20133 Milan, Italy; 5 Molecular Microbiology & Bioenergetics, Institute of Molecular Biosciences, Goethe University, 60438 Frankfurt/Main, Germany; 6 Department of Civil, Environmental and Materials Engineering (DICAM), Faculty of Engineering, University of Bologna, Bologna 40131, Italy

## Abstract

**Background:**

Horizontal gene transfer through natural transformation of members of the microbiota of the lower gastrointestinal tract (GIT) of mammals has not yet been described. Insufficient DNA sequence similarity for homologous recombination to occur has been identified as the major barrier to interspecies transfer of chromosomal DNA in bacteria. In this study we determined if regions of high DNA similarity between the genomes of the indigenous bacteria in the GIT of rats and feed introduced DNA could lead to homologous recombination and acquisition of antibiotic resistance genes.

**Results:**

Plasmid DNA with two resistance genes (*npt*I and *aad*A) and regions of high DNA similarity to 16S rRNA and 23S rRNA genes present in a broad range of bacterial species present in the GIT, were constructed and added to standard rat feed. Six rats, with a normal microbiota, were fed DNA containing pellets daily over four days before sampling of the microbiota from the different GI compartments (stomach, small intestine, cecum and colon). In addition, two rats were included as negative controls. Antibiotic resistant colonies growing on selective media were screened for recombination with feed introduced DNA by PCR targeting unique sites in the putatively recombined regions. No transformants were identified among 441 tested isolates.

**Conclusions:**

The analyses showed that extensive ingestion of DNA (100 μg plasmid) per day did not lead to increased proportions of kanamycin resistant bacteria, nor did it produce detectable transformants among the aerobic microbiota examined for 6 rats (detection limit < 1 transformant per 1,1 × 10^8 ^cultured bacteria). The key methodological challenges to HGT detection in animal feedings trials are identified and discussed. This study is consistent with other studies suggesting natural transformation is not detectable in the GIT of mammals.

## Background

Relatively few studies have examined the occurrence of horizontal gene transfer (HGT) by natural transformation in the gastrointestinal tract (GIT) of mammals under *in vivo *conditions [1-5]. It is unclear if the lack of observable competence among bacteria in the GIT is due to experimental limitations, limited occurrence in the few model bacteria examined, or due to true lack of conditions conductive for the development of competence among members of the GIT microbiota of various mammals.

There are several requirements for natural transformation to take place with non-mobile DNA in the GIT [6,7]. First, bacteria must be able to express a competent stage while in the GIT system. Numerous bacterial species present in the GIT are known to be able to develop natural competence when grown *in vitro *[8-10]. For instance, species within the genera *Bacillus, Campylobacter, Helicobacter, Lactobacillus, Neisseria, Pseudomonas, Streptococcus, and Vibrio *are known to express competence under specific laboratory conditions. However, so far only few *in vivo *studies have reported on the occurrence of bacterial competence in the GIT and those are limited to the upper GIT. The lower part of the GIT has nevertheless been suggested to be the most active site for HGT processes due to abundance of nutrients, high bacterial density and slower degradation rates of DNA [11,12].

Second, extracellular DNA of sufficient length and concentration must be present and accessible to competent bacteria. Several experimental studies have investigated the stability of DNA in the GIT of mammals ([2-4,13-20]) suggesting that minor fractions of DNA provided with different feed sources may remain in various GIT compartments. For a recent review, see Rizzi *et al. *[5]. Moreover, that minor portions of such DNA can remain of sufficient size for the acquisition of functional traits (e.g. new protein coding sequences) by competent bacteria.

Third, chromosomal DNA fragments taken up over the cytoplasmic membrane must be able to recombine with the bacterial host chromosome for stable inheritance and vertical transmission [21-23]. In general, incoming DNA must contain regions of minimum 25-200 bp in length of high similarity to the recipient genome for homologous recombination to occur [24-28]. These prerequisites are met by ribosomal gene sequences, which are sufficient in length and degree of sequence conservation for homologous recombination events [29].

Several studies have investigated conditions for natural transformation in the upper GIT. The oral cavity is the first place feed introduced DNA enters the GIT and therefore also contains the highest amount of DNA ingested. In general, a highly time-limited stability of DNA and ability to transform defined model bacteria introduced to saliva or stomach fluids has been observed *in vitro *and *in vivo *[16,30-34]. Only few studies have investigated the occurrence of natural transformation in the compartments of the lower GIT. Wilcks and colleagues [2,3] reported some persistence of introduced DNA in the GIT of germ-free/gnotobiotic or mono-associated rats, but did not detect uptake of plasmid DNA by *E. coli*, *Bacillus subtilis*, or *Streptococcus gordonii*. In another study, Nordgård *et al.*, [4] examined the ability of *Acinetobacter baylyi *colonized gnotobiotic mice and rats for potential *in vivo *transformation of bacteria with feed introduced DNA. No transformants were detected *in vivo *or *in vitro*. In the only *in vivo *study of natural transformation of bacteria present in the GIT of humans published so far, only limited persistence of food-derived plant DNA was found [1]. The DNA did not survive passage through the GIT of healthy human subjects as determined by PCR. Three out of these seven human volunteers (ileostomists) showed evidence of low-frequency HGT of plant DNA (the *epsps *transgene) to the microbiota of the small bowel, but this appeared to have occurred before feeding the experimental meal and no single microbial isolate could be obtained for further analysis. With the exception of the *in vivo *study in human described above, most published studies focusing on natural transformation in the GIT have used experimental models aimed at detecting HGT events occurring into single bacterial strains introduced into the GIT system.

**Figure 1:**
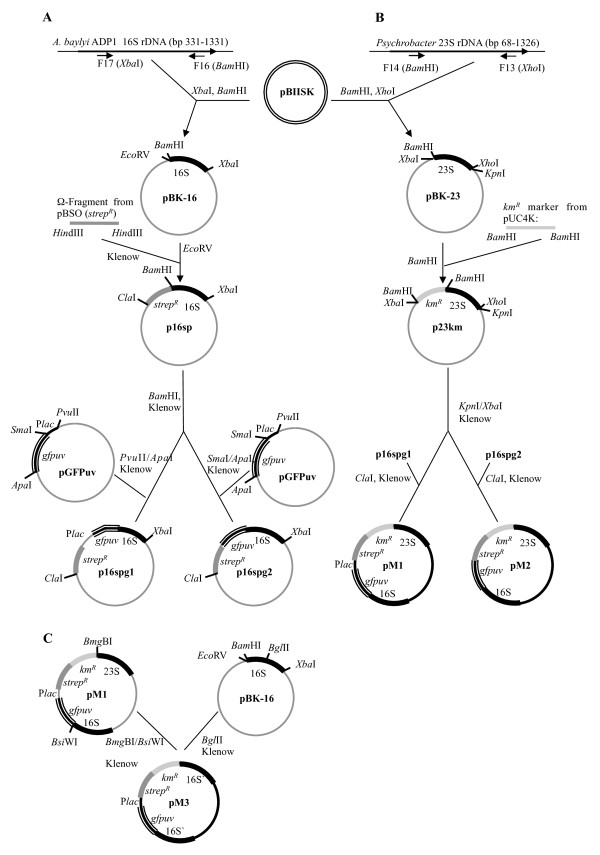
**Overview of the generation of the replacement recombination vectors pM1, pM2 and pM3**. The plasmids pM2 and pm3 were mixed with standard rat feed for the feeding trials. Km^R^; kanamycin-resistance (*npt*I); strep^R^: streptomycin resistance; gfp: *gfpuv*-gene (enhanced GFP) with native promoter. Figures are not to scale.

**Figure 2:**
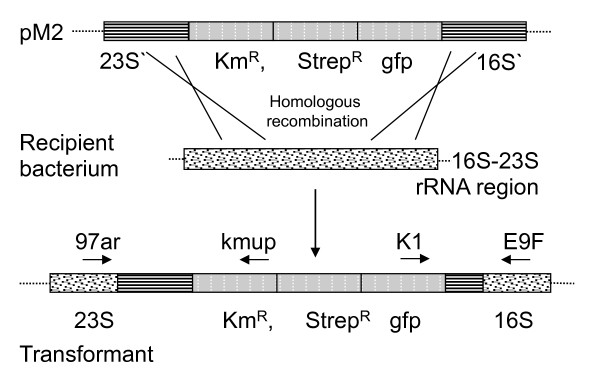
**rRNA sequence-based homologous recombination**. Schematic presentation of double homologous recombination between the pM2 and the host ribosomal 16S-23S region. The binding sites of the primers 97ar/kmup and K1/E9F used to confirm the chromosomal integration of the constructs are indicated by arrows. Km^R^; kanamycin-resistance (*npt*I); strep^R^: streptomycin resistance; gfp: *gfpuv*-gene (enhanced GFP) with native promoter; 16S: 16S rRNA sequence (1 kb) of *Acinetobacter *sp; 23S: 23S rRNA sequence (1,258 bp) of *Psychrobacter *sp. Figures are not to scale.

The aim of this study was to examine if *naturally *occurring aerobic bacteria in the GIT of rats can undergo natural transformation and recombination with feed introduced DNA. We hypothesized that regions of high DNA similarity between the introduced DNA and the indigenous bacteria in the rat GIT would lead to stable integration of two selectable marker genes (streptomycin or kanamycin resistance) based on homologous recombination. Moreover, that such events occurred at a frequency that was detectable by direct selective plating. Plasmid DNA with 16S rRNA and 23S rRNA sequences flanking the two antibiotic resistance marker genes were mixed with standard rat feed pellets (Figure 1). A double-sided homologous recombination event with sequence-similar rRNA genes in competent bacteria in the rat GIT would lead to integration of the antibiotic resistance genes (Figure 2). Recombinant-specific primer annealing sites were chosen to enable unambiguous detection of transformants carrying chromosomal integrations of the resistance genes among the overall population of bacteria recovered from the GIT.

## Results

### Natural transformation

The plasmids (purified from *E. coli*) were highly potent in generating transformants when incubated with naturally competent *A. baylyi *cells *in vitro *on LB agar plates. Transformation frequencies of above 1,4 × 10^-5 ^were obtained per 0,2 μg plasmid (data not shown): similar to previous *in vitro *studies with the same experimental setup [35,36].

To address the question whether the plasmids are also sufficient for natural transformation studies of phylogenetically distant bacteria, natural transformation assays were performed with pM1, pM2 and pM3 using ~30 different marine isolates as potential recipients. These studies revealed the ability of different Gammaproteobacteria and Actinobacteria to be naturally transformed with the three DNA constructs, even in the presence of significant differences in 16S rRNA identity (Table 1).

**Table 1:** Natural transformation of environmental isolates of bacteria^a^

**Transformable isolate**^**b**^	Phylogenetic position	Plasmids tested	**16S-rRNA gene identity**^**c**^
*Marinobacter *sp.	Gammaproteobacteria;	pM1, pM2,	77
	*Alteromonadales*	pM3	
*Kocuria rosea*	Actinobacteria;	pM2	79
*Photobacterium*	Gammaproteobacteria;	pM1, pM2,	86
*phosphoreum*	*Vibrionales*	pM3	
*Psychrobacter*	Gammaproteobacteria;	pM1, pM2,	96
*marincola*	*Pseudomonadales*	pM3	

^a^Outcome of a study examining approximately 30 isolates obtained from three sources (Mediterranean, Celtic and north Atlantic seas).

^b^Transformants were species identified by 16S sequencing and confirmed by selective plating and PCR. The screen for transformability did not aim to be quantitative, however, the overall CFU numbers indicated a transformation frequency of ~ 10^-5 ^to 10^-6 ^transformants per total CFU. The utility of the plasmids for transformation studies was analyzed by use of marine isolates since the recombinant plasmids for genomic integration carried rRNA genes from a marine *Psychrobacter *isolate.

^c^Percentage identity to 16S rRNA of *Psychrobacter*.

### Total CFU and resistant CFU in the gut contents

The total CFU and the number of resistant CFU in samples from the stomach, small intestine, cecum and colon of rats were determined after the 4 days of plasmid exposure. The mean numbers of bacteria in the different compartments are presented in Table 2. The bacterial counts varied between individuals, for instance, in the stomach, the total CFU ranged from < 1 × 10^3 ^to 6 × 10^5^, in the small intestine the CFU varied from < 10^3 ^to 2 × 10^7^. Total CFU obtained for material sampled from the cecum and colon were lower than expected, ranging form < 1 × 10^6 ^(not quantifiable due to dilution error, most of the individuals) to up to 4 × 10 ^7^. Variability in CFU numbers is expected due to different amounts of food material present in the colon of each rat; reflecting differences in activity, eating pattern and digestion.

**Table 2:** CFUs obtained from the gastrointestinal tracts of rats, with a normal microbiota, consuming standard feed with added DNA over 4 days (100 μg/day)

Treatment	**Total CFU/g faeces Range between animals**^**b**^	Resistant CFU/g faeces Range between animals	Resistant CFU Mean (total no.)	**Detection limit**^**a**^
No DNA in feed (n = 2)				
Stomach	1,4 × 10^5 ^- 5,0 × 10^5^	0	0 (0)	-
Small	1,8 × 10^5 ^- 1,8 × 10^5^	0	0 (0)	-
intestine				
Cecum	< 10^6^	4-4	4 (8)	-
Colon	< 10^6^	4-5	4,5 (9)	-
With DNA in feed (n = 6)				
Stomach	< 10^3 ^- 6,0 × 10^5^	0-180	30 (181)	< 8,3 × 10^-7^
Small	< 10^3 ^- 1,5 × 10^7^	0-36	6 (36)	< 4,3 × 10^-8^
intestine				
Cecum	< 10^6^	0-84	24 (146)	< 1,0 × 10^-6^
Colon	< 10^6 ^- 3,8 × 10^7^	0-72	13 (78)	< 1,1 × 10^-8^
Sum (6 rats)	1,1 × 10^8^	0-180	441	< 8,8 × 10^-9^

^a^All the 441 colonies growing on selective-agar plates were tested by PCR for possible uptake of the plasmid DNA and none were found positive. The detection limit is therefore calculated as the reciprocal of the total CFU (summarized for each compartment for all 6 rats); and given as an absolute number. As seen from the range of total CFU obtainable per individual animal, the detection limit can vary 1000-fold. Specifically, the summarized CFU numbers for the compartments of the six DNA feed rats were 1,2 × 10^6 ^(stomach), 2,3 × 10^7 ^(small intestine), < 10^6 ^(cecum) and 9,0 × 10^7 ^(colon).

^b^Despite preliminary studies to quantify the expected total CFUs, the number of CFU in the cecum and colon fell in several cases below the expected concentration/plate dilutions made. Hence the total CFU number could not be determined.

CFU numbers of resistant colonies determined by plating on selective media containing streptomycin and kanamycin were very low. In most animals we could not detect culturable resistant bacteria; with a detection limit of 1 CFU per gram material. However, from some compartments and individuals a few colonies emerged on the selective media (see Table 2). A total of 441 isolates were collected from plates inoculated with GIT material obtained from exposed rats, and 17 isolates from GIT material obtained from non-plasmid exposed rats. All isolates growing on selective media were picked and frozen in LB media with 15% glycerol for further characterization.

### PCR analyses of antibiotic resistant colonies

None of the 441 antibiotic resistant bacteria recovered from the GIT content of DNA exposed rats produced a positive PCR signal. The total amount of bacteria plated on selective media from the six exposed individuals exceeded 1 × 10^8 ^bacteria. Thus, less than 1 antibiotic resistance gene transfer event was observed per 1,1 × 10^8 ^culturable bacteria. The transformation frequency of the culturable bacterial fraction with the feed added plasmid was therefore less than 8,8 × 10^-9 ^transformants per culturable bacteria.

## Discussion

Natural transformation of microbial communities in the GIT requires the presence of bacteria that express competence in gut locations where DNA is present and accessible. Moreover, those competent bacteria must be able to integrate and vertically transfer the DNA over subsequent generations (i.e. through linkage to a replicating unit). Finally, no significant absolute or relative fitness reductions should be experienced by the transformed cells [37]. The model system presented here was optimized such that the requirements for stable inheritance were met through providing the target DNA with flanking regions sharing homology to bacterial rRNA genes harboured by a subset of the bacteria present in the GIT of rats. Table 3 lists the bacterial species with less than 25% DNA sequence divergence at the 16S or 23S loci.

**Table 3:** Bacterial genera with species that are less than 25% divergent at the 16S or 23S loci compared to the DNA sequences present in plasmids pM2 and pM3

**pM2 **(with 16S rRNA from *Acinetobacter*)	*Aneurinibacillus, Bacillus, Brevibacillus, Caloramator, Caryophanon*,
	*Clostridium, Desulfotomaculum, Escherichia, Eubacterium*,
	*Filobacillus, Halobacillus, Hespellia, Listeria, Marinibacillus*,
	*Oceanobacillus, Paenibacillus, Pelospora, Planococcus*,
	*Planomicrobium, Ruminococcus, Salinicoccus, Syntrophomonas*,
	*Thermobrachium, Virgibacillus*.
**pM3 **(with 23S rRNA from *Psychrobacter*)	*Acidithiobacillus, Acidovorax, Acinetobacter, Aeromonas*,
	*Aggregatibacter, Alcanivorax, Alicycliphilus, Alicyclobacillus*,
	*Aliivibrio, Alkalilimnicola, Alkaliphilus, Allochromatium, Alteromonas*,
	*Anaerococcus, Anaerofustis, Anaerostipes, Anaerotruncus*,
	*Anoxybacillus, Aromatoleum, Azotobacter, Bacillus, Baumannia*,
	*Blautia, Brevibacillus, Buchnera, Burkholderia, Butyrivibrio*,
	*Cardiobacterium, Catonella, Cellvibrio, Chromobacterium*,
	*Chromohalobacter, Citrobacter, Clostridium, Colwellia, Comamonas*,
	*Congregibacter, Coprococcus, Cronobacter, Cupriavidus*,
	*Dechloromonas, Delftia, Desulfitobacterium, Desulfotomaculum*,
	*Dichelobacter, Dickeya, Dorea, Edwardsiella, Eikenella, Enterobacter*,
	*Erwinia, Escherichia, Ethanoligenens, Eubacterium, Exiguobacterium*,
	*Faecalibacterium, Ferrimonas, Finegoldia, Francisella, Gallionella*,
	*Gemella, Geobacillus, Haemophilus, Hahella, Halomonas*,
	*Halorhodospira, Halothiobacillus, Heliobacterium, Herbaspirillum*,
	*Hydra, Idiomarina, Kangiella, Kingella, Klebsiella, Lachnospiraceae*,
	*Laribacter, Legionella, Leptothrix, Listeria, Lysinibacillus*,
	*Macrococcus, Marinobacter, Methylibium, Methylobacillus*,
	*Methylococcus, Methylophaga, Methylotenera, Moraxella, Neisseria*,
	*Nitrococcus, Nitrosomonas, Nitrosospira, Oceanobacillus*,
	*Oribacterium, Oxalobacter, Paenibacillus, Pantoea, Parvimonas*,
	*Pasteurella, Pectobacterium, Pelotomaculum, Peptoniphilus*,
	*Photobacterium, Photorhabdus, Polaromonas, Proteus, Providencia*,
	*Pseudoalteromonas, Pseudomonas, Pseudoxanthomonas*,
	*Psychrobacter, Psychromonas, Ralstonia, Rhodoferax, Roseburia*,
	*Ruminococcus, Saccharophagus, Salmonella, Serratia, Shigella*,
	*Shuttleworthia, Sideroxydans, Simonsiella, Staphylococcus*,
	*Stenotrophomonas, Subdoligranulum, Symbiobacterium*,
	*Syntrophobotulus, Syntrophomonas, Syntrophothermus, Teredinibacter*,
	*Thermaerobacter, Thiomicrospira, Thiomonas, Variovorax, Vibrio*,
	*Wigglesworthia, Xanthomonas, Xenorhabdus, Xylella, Yersinia*.

The *in vivo *model system used also included a selectable marker system (*npt*I, *aad*A) that facilitated unambiguous detection of HGT among the diversity of the culturable bacterial populations. Although the model system chosen attempted to circumvent some natural barriers to HGT (introduced DNA sequence similarity) and model based limitations (strong positive selection of transformants, and high levels of DNA exposure to natural bacterial communities) no transformants were observed among the 6 rats and approx. 10^8 ^cultivable, aerobic bacteria examined. Several factors relevant to these findings may explain the lack of observable uptake of plasmid DNA in this study:

i) Natural transformation does not occur in the GIT of rats due to lack of competence-expressing bacteria or lack of access to extracellular DNA for competent bacteria.

ii) Natural transformation occurs at frequencies below the levels detectable within a limited number of aerobic bacteria per animal GIT and days of DNA exposure. The various parts of the GIT support different inoculum densities [12]. Although total CFU numbers were at reasonable levels in the various gut compartments in rats (10^3^-10^7 ^CFU per gram GIT content), these population sizes may be too small to enable identification of rare HGT events in individual, single GITs [4,38]. The detection limit per rat and GIT compartment is thus 1 transformant per 10^3 ^to 10^7 ^bacteria, in our study. A detection limit of 1 transformant per 1 × 10^8 ^bacteria is reached when the overall number of CFU tested is summarized for the 6 rats. Transformable bacterial species may nevertheless express competence below this frequency range and screening of larger bacterial population sizes should be considered for consistent detection or rare HGT events. The number of species in the GIT of rats that are expected to be competent based on *in vitro *data is only a fraction of overall population size and diversity. It can be estimated that it would be necessary to sample the combined bacterial populations of the GIT content of 60 to 6 000 rats to identify bacterial transformants produced at lower frequencies of 10^-10 ^to 10^-13^.

iii) Natural transformation is limited in frequency due to the low concentrations or lack of accessibility of DNA substrates in the different compartments of the digestive tract. Although a continuous supply of DNA was ensured by daily administration of high amounts (100 μg plasmid DNA), purified DNA is known to rapidly fragment *in vivo*. However previous studies have shown that minor proportions of orally ingested plasmid DNA persist in a biologically active form in the GIT of rats [2,3], and 1-2% of plasmid and bacteriophage DNA can survive passage through the mouse GIT and be detected in the faeces [13-15]. The latter study detected size ranges from a few hundred bp up to about 1700 bp. Similar observations of short fragment size have also been reported in other animal experiments involving fish, poultry, pig, sheep, cattle and humans [16-19,32,39-41]. See Rizzi *et al. *[5], for a comprehensive review. However, almost all of these studies have biochemically analyzed persistence and degree of fragmentation after recovery of DNA from the gut. So far, little information is available on the extent that DNA fragments present in various gut compartments are physically accessible to bacteria as templates for natural transformation [4,5]. The study by Nordgård et al. [4] indicates that gut contents may be inhibitory to natural transformation. The study examined the effects of the GIT content of both normal microbiota rats and germfree mice in *in vitro *transformation assays of competent cells of *A. baylyi*. The study showed that the presence of both types of gut contents was inhibitory to transformation [4]. Only purified DNA added to cecum and large intestine content samples from germfree mice was able to transform *A. baylyi *at low frequencies *in vitro*. The sharply reduced DNA uptake frequencies also observed in the presence of sterile gut material from mice indicate that microbially produced DNA nucleases were not responsible for the absence of observable transformation. Bacterial nucleases have also in other studies been found to play a minor role in DNA degradation in the GIT [2,3,42]. Thus, host nucleases or other macromolecules present in the GIT may inhibit natural transformation.

iv) Natural transformation occurs in the GIT but is not observed in our model systems due to other technical limitations. These limitations can include the possibility that bacterial species present in the gut only express competence as a response to certain host physiological conditions or certain (feed) nutrition sources not adopted in our feeding protocol. Moreover, it cannot be excluded that the plasmid DNA used for our transformation study *in vivo *was not optimal for DNA uptake among all the relevant competent bacterial species in the gut. The DNA similarity, present on the plasmids used, is highest to the *Acinetobacter *and *Psychobacter *genera. However, the *in vitro *experiments with some marine isolates confirmed the broader applicability of these vectors for natural transformation studies. To our knowledge the results obtained from the *in vitro *study of the marine isolates represent the first report of natural transformation of members of the genera *Photobacterium*, *Marinobacter *and *Psychrobacter *(Gammaproteobacteria) and the genus *Kocuria *(Actinobacteria). These observations suggest that the constructed vectors can be used to detect novel transformable bacteria from various environmental samples. As most bacteria have multiple copies of rRNA genes [43], a lethal effect of an integration of the marker genes into a single rRNA locus is not expected.

Finally, molecular evidence indicates that as little as 10% and up to 40-50% of the GIT population can be identified by differential plating methods [44]. The inability to isolate the major fraction of the microorganisms in the GIT, and importantly the obligate anaerobic proportion, on agar media could also contribute to the failure to detect transformants in our model system. However, it is emphasized that most GIT bacteria that are of high concern in the context of resistance development do belong to the culturable fraction (e.g. the *Enterobacteriaceae*). The lack of provision of an immediate selective advantage to the transformants may also have given the net result that rare transformants failed to survive and to expand to numbers that were sufficient to allow their detection [37].

## Conclusions

Natural transformation occurring among members of the bacterial community in the lower GIT remains to be demonstrated. Most previous studies have examined natural transformation of single bacterial species introduced into the GIT of various mammalian species. The model system presented here allowed a subset of the aerobic microbial community to be tested for competence development; however, transformants were not detected among the 6 rat GITs and 10^8 ^bacteria tested; suggesting a transformation frequency below 8.8 × 10^-9 ^(for the combined rat samples). As discussed here, small rodent models may harbour too few culturable bacteria per individual and gut compartment to allow realistic detection limits of natural transformation events in the lower GIT. The design of future studies must consider the opportunities and limitations inherent in the population sizes of culturable aerobic and anaerobic bacteria in the model organism used, the transformable fraction and level of competence expressed among these, and the DNA exposure rates expected in the various GIT compartments

## Methods

### Plasmids

Three chromosome integration vectors, pM1, pM2 and pM3 were derived from the *E. coli *vector pBIISK (*ori*V of pMB1) (Figure 1). pM1 (9500 bp) and pM2 (9338 bp) both carry the 16S rRNA gene (bp 331-1331) from *Acinetobacter baylyi *ADP1 and the 23S rRNA gene (bp 68-1326) from a marine *Psychrobacter *isolate. The 16S rRNA gene was amplified with primer pair F16 and F17, and the 23S rRNA gene was amplified with primer pair F13 and F14. The rRNA sequences flank genes encoding the green fluorescent protein (GFP) and streptomycin and kanamycin resistance. The two resistance markers can be used for selection of transformants. The *gfp *gene enables detection of transformants but was not used throughout this study since transformants were already obtained by selection alone. In pM3 (8281 bp) the resistance genes and *gfp *gene are flanked by two 16S rRNA gene sequences (bp 362-693 and bp 694-1331) (Figure 1). The plasmids were maintained in *E. coli *TOP10 cells (Invitrogen) grown on LB agar containing streptomycin (50 μg/ml) and kanamycin (100 μg/ml). Plasmid DNA was isolated using the Qiagen Plasmid Maxi Kit (Qiagen, Germany) following the manufacturer's protocol and quantified by Nanodrop ND-1000 (Nanodrop Technologies) prior to dilution and mixing in feed. The integrity of the plasmids was also confirmed by agarose gel electrophoresis.

### Bioinformatic analyses

The level of similarity between the 16S and the 23S rRNA gene fragments inserted into the pM2 and pM3 plasmids with known DNA sequences was determined. The partial nucleotide sequence (331-1331 bp) of the 16S rRNA gene of *A. baylyi *DSM14961 type strain (EF611407) and the partial nucleotide sequence (68-1326 bp) of the 23S rRNA gene of *Psychrobacter faecalis *strain DSMZ 14664 (HM236417) were used for BLASTN retrieval. The search was limited to the type strain bacteria of the most common genera found in rat and in mouse GIT system [45-49]. For the 16S rRNA fragment, the BLASTN search was to 101 different bacterial species, while for the 23S rRNA fragment it included 302 different bacterial species. The maximum target sequences parameter was set to 500.

### Natural transformation

Cells of the naturally competent *A. baylyi *strain BD413 were exposed to the plasmids (pM2 and pM3) to confirm that the purified plasmids were able to transform a known naturally competent bacterium at high frequencies. For the *in vitro *filter transformation assays, 0.2, 2, or 20 μg of purified plasmid DNA (Qiagen Miniprep kit, Germany) was mixed with 100 μl of bacterial cells, and incubated on nitrocellulose filters on LB agar plates for 24 h to allow binding and uptake of DNA and replacement recombination events of the ribosomal gene sequences (Figure 2.), before serial dilutions and selective plating on LB agar as described by Ray and Nielsen [50]. To confirm the broader functionality of the plasmids for natural transformation, available marine isolates of the Gammaproteobacteria, Actinobacteria and Alphaproteobacteria were exposed to pM1, pM2 and pM3. Purified plasmid DNA (1 μg) was linearized with *Not*I and mixed with 100 μl of overnight culture of a marine isolate and incubated on nitrocellulose filters on marine broth (MB) agar (18.5 g marine broth, 10 g NaCl and 15 g agar per liter, pH 7.6) at room temperature for 24 to 48 h (until growth was visible). After serial dilutions of the cells with saline, selective plating was done on MB agar containing 100 μg/ml streptomycin and 100 μg/ml kanamycin. Transformation was confirmed by selective plating and PCR using primers E9F and K1 for amplification of the 16S rRNA gene and *gfp*, and primers kmup and 97ar for amplification of *npt*I and 23S rRNA genes (Table 4 and Figure 2).

**Table 4:** PCR primers used in this study

Target	Name	Size	Primer sequence (5'-3' direction)	References
Insertion of pM2	KanR-F	800 bp	ATC GCA GTG GTG AGT AAC	This study,
	ITSR-Eub		GCC AAG GCA TCC ACC	[51]
	16S-926 F	850 bp	AAA CTT AAA TGA ATT GAC GC	[52]
	GFP-R		TTC TTT TGT TTG TCT GCC ATG ATG TAT	This study
Insertion of pM3	16S-PrMir530F	1030 bp	GTG CCA GCA GC GCG G	This study
	GFP-F		ATA CAT CAT GGC AGA CAA ACA AAA GAA	This study
16S	10 F	900 bp	AGT TTG ATC ATG GCT CAG ATT G	[53,54]
	907R		CCG TCA ATT CHT TTR AGT TT	
16S of *A. baylyi*	F16	1000 bp	ACT AGC GGA TCC GAC TTC	This study
	F17		CCA GAC TTC TAG AGG AGG C	
23S of *Psychrobacter*	F13	1258 bp	CGT TGG ACT CGA GCC CTT G	This study
	F14		CACATGGTGGATCCCTTGG	
16S *gfp*	E9F-K1	1500 bp	GAG TTT GAT CCT GGA TCA	This study
			TTG GCC ATG GAA CAG GTA	
km^R ^23S	Kmup-97ar	2748 bp	GTA TGA GTC AGC AAC ACC TTC	This study
			CGC TTA GAT GCT TTC AGC	

### Rat feed preparation

Food pellets were made by mixing 80 ml sterile water with 200 g of standard rat feed powder AIN-93 (Scanbur BK AS, Norway) [55]. After solidification overnight at 4°C, the mass was cut into 1.5 × 1.5 × 1.5 cm feed pellets and left on a tray covered with baking paper to dry overnight in a sterile hood at RT. The pellets were kept at -20°C until used. A total of 100 μg plasmid DNA (pM2 and pM3 at equal amounts) was pipetted into the dry feed pellet. Control rats received the same pellets, but without DNA. Previous studies have shown that the feed source is free of DNA and that DNA mixed into the pellet does not degrade or lose its ability to transform bacteria over a 72 h incubation period at RT [56].

### Feeding trial

Wistar rats (Mol:WIST Han, M&B Denmark), bred at the rat facility of the Animal Department of the University of Tromsø, Norway were used. The rats (200 ± 20 grams) were referred to two different groups. Group one consisted of two rats: one female and one male and group two: three females and three males. Group one was given the pellet meal only (contained no DNA; negative control group), while group two was given the target meal (feed pellets with added plasmid DNA). The total amount of plasmid DNA ingested per rat in group two was 100 μg per day for four days (50% pM2 and 50% pM3). Plasmid DNA was provided as a purified DNA extract; with no restriction enzyme treatment. The rats were killed in a CO_2 _chamber 4 days after starting on the target meal, and immediately before sampling of contents from the different GI compartments (stomach, small intestine, cecum and colon). The experiments/housing procedures were approved by The National Animal Research Authority, Norway.

### Enumeration of bacterial cells

Before starting the feeding trials, the overall background level of antibiotic resistance to streptomycin and kanamycin were determined for faeces from rats kept at the Animal facility used for the feeding trial. No colonies emerged on the selective plates (data not shown); indicating a low level of background resistance among the aerobic population and suitability of streptomycin or kanamycin in identifying bacterial transformants.

The content from the different sampling sites (stomach, small intestine, cecum and large intestine) were plated on LB agar-plates. Total CFUs (colony forming unit) per gram were determined under aerobic conditions on non-selective plates (100 μl of ten-fold dilutions made in saline), and on plates with streptomycin or kanamycin (both 50 μg/ml) for transformant CFU per gram. Approx. 20 transformant-selective agar plates were used per sample (of approx. 1 g) to ensure sufficient dispersal of gut material per plate and to prevent bacterial growth on insoluble feed material. CFU were determined after incubation at 37°C for 46 h. The transformation frequencies are given as the number of CFU growing on transformant-selective plates divided by the number of CFU on non-selective plates. The detection limit is given as the reciprocal of the number of CFU on non-selective plates. The colonies grown on the selective plates were picked and kept at -20°C in LB medium containing 10% glycerol and kanamycin or streptomycin at 50 μg/ml until further analysis. The total number of bacterial cells tested for potential uptake of added plasmid DNA was calculated as the sum of the numbers of bacteria (CFUs) obtained under aerobic conditions on non-selective plates for the 6 treated rats combined (1,1 × 10^8 ^CFUs); as an equal or higher amount of sample was also plated on the selective media for growth of transformed cells. Thus, the detection limit represents the potential for transformation occurring in the GIT of a rat population consisting of 6 individuals.

### Bacterial DNA isolation and PCR amplification

DNA was isolated from colonies that emerged on the selective agar plates with the GenElute Bacterial Genomic Kit (Sigma) following the manufacturer's protocol. Isolated DNA was quantified with a Nanodrop ND-1000 (Thermo, USA). The DNA from single, re-streaked colonies was subjected to PCR analysis of the bacterial 16S rRNA gene to confirm the general absence of PCR inhibitors. The reactions were performed in a total volume of 25 μl containing the following: 0,75 μl of each primer at 10 μM, 12,5 μl HotStarTaqMix (Qiagen, Germany), 10 μl water and 1 μl bacterial DNA template (100 ng/μl). Primers and cycling conditions used are listed in Tables 4 and 5. The PCR products were run on 1% agarose gels before visualization.

**Table 5:** PCR cycling conditions

Primers	Initial Denaturation	Denaturation	Annealing Elongation	Cycles	Terminal Elongation
KanR F and ITSR-Eub R	95°C/5 min	95°C/30 s	54°C/25 s 72°C/70 s	35	72°C/7 min
16S-926 F and GFP R:	95°C/5 min	95°C/30 s	53°C/40 s 72°C/90 s	35	72°C/7 min
16S-Pr.mir-530 F and GFP F	95°C/5 min	95°C/30 s	63°C/25 s 72°C/70 s	35	72°C/7 min
16S	94°C/15 min	94°C/4 min	50°C/45 s 72°C/1 min	5	
16S (cont.)		92°C/45 s	55°C/45 s 72°C/1 min	30	72°C/7 min
13 F-14 F	97°C/5 min	97°C/30 s	50°C/45 s 72°C/1.5 min	30	72°C/7 min
16 F-17 F	97°C/5 min	97°C/30 s	50°C/45 s 72°C/1 min	30	72°C/7 min
E9F-K1	97°C/5 min	97°C/30 s	56°C/45 s 72°C/1.5 min	30	72°C/7 min
Kmup-97ar	97°C/5 min	97°C/30 s	56°C/45 s 72°C/2.5 min	30	72°C/7 min

To determine if the emerging antibiotic resistant bacteria arose from recombination (transformants), primers targeting different regions of the two plasmid constructs were used (Table 4). For each resistant bacterial colony obtained, PCR was done to define if a fragment of the plasmid (pM2 or pM3) was inserted into the genome. The reactions were performed in a total volume of 50 μl containing the following: 1 μl of each primer at 10 μM, 25 μl DYNAzyme™ II PCR Master Mix (Finnzymes Oy, Finland), 18 μl ddH_2_O and 2 μl DNA template (100 ng/μl). Negative PCR setup controls (no DNA template), negative rat controls (received no DNA in feed) and positive controls (plasmid dilution series) were included in each PCR set-up. Primers and cycling conditions are listed in Tables 4 and 5.

## Competing interests

The authors declare that they have no competing interests.

## Authors' contributions

LN participated in the design, coordination, analysis, and drafting of the manuscript. LB and NR participated the molecular and microbiological experiments and analyses, TT participated in the design and drafting of the manuscript. BA designed the plasmids, contributed the data on the marine isolates, and revised the manuscript. KMN contributed to the design, coordination, analysis, and drafting of the manuscript. All authors read and approved the final manuscript.
